# Estimation of Cardiorespiratory Fitness Without Exercise Testing: Cross-Validation in Midlife and Older Women

**DOI:** 10.1089/whr.2020.0045

**Published:** 2020-12-28

**Authors:** Jody L. Clasey, Anita M. Adams, Paul J. Geiger, Suzanne C. Segerstrom, Leslie J. Crofford

**Affiliations:** ^1^ Department of Kinesiology and Health Promotion, Center for Clinical and Translational Science, University of Kentucky, Lexington, Kentucky, USA.; ^2^ Department of Psychology, University of Kentucky, Lexington, Kentucky, USA.; ^3^ RTI International, Durham, North Carolina, USA.; ^4^ Department of Medicine, Vanderbilt University Medical Center, Nashville, Tennessee, USA.

**Keywords:** cardiorespiratory fitness (CRF), physical activity, women's health

## Abstract

**Background:**

Cardiorespiratory fitness (CRF) is associated with important health risk outcomes, including the development of Type 2 diabetes and cardiovascular disease. Measures of maximal or peak oxygen consumption (VO_2_) are the typical criterion methods for determining CRF; however, in clinical settings, these measures are impractical.

**Methods:**

We validated a clinically derived estimate of CRF against predicted maximal VO_2_ in a sample of healthy, midlife and older adult women (*n* = 188). Women completed a clinic evaluation (including treadmill testing), daily diaries about their physical activity, and additional clinical scales. Two models were tested. The first model calculated estimated cardiorespiratory fitness (eCRF) using assigned weights and regressed eCRF on predicted cardiorespiratory fitness (pCRF). The second model used sample-specific, empirical weights. Both models were tested twice, once with retrospective and once with daily diary physical activity reports.

**Results:**

The model accounted for 34% of the variance in pCRF when using assigned weights and 41% of the variance in pCRF when using empirical weights. For age, body mass index, and resting heart rate, assigned and estimated weights were similar, but estimates for physical activity differed. There was little improvement in model fit between retrospective and daily diary measurements of physical activity when either assigned (*R*^2^ = 0.32) or fitted weights (*R*^2^ = 0.40) were used.

**Conclusions:**

Midlife and older women's CRF can be estimated from routinely collected clinical measures, demonstrating their utility.

## Introduction

Measurement of cardiorespiratory fitness (CRF) is typically performed using ventilatory gas exchange, with the volume of oxygen consumed most often expressed relative to body weight per minute (VO_2_) either at submaximal or maximal exertion.^1^ Using measures obtained from progressive submaximal graded exercise testing (GXT), the maximal oxygen uptake and utilization (VO_2max_) can be estimated using population-specific equations.^2^

Low CRF affects important health outcomes, including metabolic syndrome, type 2 diabetes, cardiovascular disease, cancer, and mortality.^3–12^ A landmark study demonstrated that objectively measured CRF was strongly associated with mortality.^5^ For women (*n* = 3,120), adjusted all-cause mortality declined from 39.5 per 10,000 person-years to 8.5 per 10,000 person-years (slope = −5.5) from the least fit to most fit quintiles.^5^ These trends remained after adjustments for age, smoking, blood pressure, cholesterol, blood glucose, family history of cardiovascular disease, and follow-up intervals. Lower risks for mortality associated with cardiovascular and cancer were also seen in those with higher levels of CRF. The attributable risk of low CRF to mortality was 48.4% in women.^5^ In a larger follow-up study from this same group, low CRF and smoking were the only independent risk factors for mortality in women.^6^

In a recent study, apparently, healthy women with low CRF (*n* = 620) were 28%, 34%, and 34% more likely to die from all causes, cardiovascular disease, and cancer, respectively, than the high CRF group (*n* = 492).^12^ Many previous studies were conducted either exclusively or predominantly in men; however, one study in asymptomatic women from the St. James Take Heart Project also demonstrated a reduction in Framingham risk score-adjusted mortality risk of 17% for each metabolic equivalent (MET) of exercise capacity.^4–6,11–13^ A recent statement from the American Heart Association affirms that data have firmly established that low levels of CRF are associated with a high risk of cardiovascular disease and all-cause mortality.^14^ Health benefits from improvements in CRF are most apparent in those at the low end of the continuum.

In most clinical settings, however, GXT (with or without accompanying VO_2_) is impractical. Equations that provide estimates of CRF (eCRF) using routinely collected clinical information, including gender, age, body mass index (BMI), resting heart rate (RHR), and self-report physical activity, have been developed.^15,16^ One of the largest of studies to develop weighted equations estimating CRF used three large datasets.^15^ The National Aeronautics and Space Administration (NASA)/Johnson Space Center cohort involved 1,458 male and 401 female employees with mean ages of 46 and 40 years, respectively.^17–19^ The participants were well educated, higher in socioeconomic status, and predominately non-Hispanic whites. The data were collected between 1971 and 2002. The Aerobics Center Longitudinal Study (ACLS) cohort consisted of 35,826 men and 10,364 women 20–70 years of age from the Cooper Clinic in Dallas, TX.^5,6^ Again, the participants were well educated, of middle and higher socioeconomic status, and mostly non-Hispanic white adults. The English 1990 Allied Dunbar National Fitness Survey (ADNFS) participants were 853 men and 853 women, also between 20 and 70 years of age and representative of the 30 parliamentary constituencies.^20^ Self-reported physical activity was assessed using different instruments in these three studies, but was collapsed into five categories that were then applied across the datasets.^15^

The Norwegian Nord-Trøndelag Health (HUNT) study (*n* = 15,217; 52.1% women) found that high eCRF was associated with a 26% reduction in all-cause mortality relative to low eCRF.^16^ eCRF has thus been shown to be one of the strongest predictors of mortality.^12,21^ In women, the association between low CRF for all-cause and cancer-related mortality may be stronger than that in men.^12^ Therefore, validation of eCRF approaches specifically for women is important for advancing clinical research on the relationship between fitness and health. The aim of this study was to determine the validity of estimating CRF in a sample of healthy midlife and older women using easily obtainable clinical information.

## Materials and Methods

### Participants

The study sample was drawn from the Daily Activity and Health in the Lives of Adult Women (DAHLiA) study. DAHLiA was approved by the University of Kentucky Institutional Review Board. For this analysis, data from 188 of 200 women in the DAHLiA cohort who completed treadmill testing were used. The remainder of the cohort were missing data and thus not included in this analysis because they failed to meet submaximal exercise criteria during the treadmill test (*n* = 3), there was equipment failure (*n* = 8), or they had an outlying value (insufficient difference between estimates to accurately calculate the slope in the submaximal exercise test; *n* = 1). These women were 99% non-Hispanic white adults who were well educated, with an average of 16.7 years of education (range 14–22 years). Additional descriptive characteristics of this subsample are given in Table 1.

**Table 1.** Sample Characteristics

Variable	Mean	SD	Range
Age (years)	62.2	6.3	50.1–76.0
BMI (kg/m^2^)	27.0	4.9	16.6–39.8
RHR (beats/minute)	73.4	10.4	51.7–120.0
Percentage of predicted maximal HR achieved (%)	86.2	6.2	74.4 – 106.9
VO_2_max (METs)	7.9	2.0	3.4–13.8
Physical activity category (CHAMPS)			1–5
1	1%		
2	6%		
3	16%		
4	18%		
5	59%		
Physical activity category (diary) (%)			1–5
1–2	50%		
3	42%		
4	5.5%		
5	2.5%		

BMI, body mass index; CHAMPS, Community Healthy Activities Model Program for Seniors; HR, heart rate; MET, metabolic equivalent; RHR, resting heart rate; SD, standard deviation.

### Procedure

Women 50–75 years of age in a seven-county area were recruited for DAHLiA from the Kentucky Women's Health Registry. Additional eligibility criteria were BMI ≤40, no pacemaker or serious cardiac condition (including blood pressure >200/100 mmHg), no serious medical conditions or mental disorders, and no oral or inhaled corticosteroids in the 3 months before enrollment. Women also had to be able to exercise on a treadmill (*i.e*., they had no physical conditions that limited their mobility). Eligible and interested women were enrolled and provided written informed consent. They underwent a clinic evaluation, including a physical examination, resting electrocardiogram (ECG), blood draw, and body composition measures, followed by a progressive graded exercise (treadmill) with continuous VO_2_ test. They then completed further evaluation through seven daily diaries completed at home, repeated every 3 months for 2 years. An interviewer administered additional scales in person at the end of each diary period. These data are taken from the clinic evaluation and the first daily diary period. Women were compensated $50 for the clinic visit, $25 for each diary period, and $25 for completing all diaries within the specified time on every diary day in that period. All study procedures were approved by the University of Kentucky Institutional Review Board.

### Measures

#### Anthropometric and BMI

Standing height and body weight were determined for each subject at the clinic visit using a wall-fixed stadiometer (Seca Statiometer Pat. No. 4694581) and a calibrated scale (Teraoka D1–10; Singapore), respectively, while wearing lightweight clothing and no shoes. BMI was calculated as kg/m^2^.

#### Resting heart rate

RHR was taken as the mean heart rate from a 10-minute seated ECG collected with a Biopac EKG100C unit connected to a Biopac M150 data acquisition system. ECGs were edited for abnormal beats using Mindware (Galeta, OH) HRV software according to accepted standards.^22^

#### Cardiorespiratory fitness

Each subject performed a submaximal GXT (3-minute progressive speed and grade) on a treadmill using an indirect calorimetry testing system with integrated ECG (SensorMedics Vmax Encore, CareFusion Corporation, San Diego, CA). During the tests, continuous measurements of oxygen consumption were recorded, cardiovascular measurements were monitored, and verbal encouragement was provided throughout. At the final 30 seconds of each stage, heart rate, blood pressure, and rating of perceived exertion were taken and recorded. The test was terminated at the end of a workload stage, eliciting a heart rate response between 115 and 150 bpm. Although this target heart rate range was specified by the chosen submaximal protocol and subsequent equation for calculating predicted maximal oxygen uptake (VO_2_max), additional termination criteria of volitional fatigue and/or abnormal ECG or excessive increases in blood pressure were included. Following the GXT, the VO_2_max was estimated using the following formula^23^:

$$VO_{2}max=SM2+b\left(HRmax-HR2\right)$$

where HRmax = predicted maximal heart rate.

$$b=\left(SM2-SM1\right)∕\left(HR2-HR1\right)$$

where SM1 and SM2 = the VO_2_ measure and HR1 and HR2 = the heart rate of the corresponding final two workload stages.

#### Physical activity

Physical activity was operationalized using the interviewer-administered Community Healthy Activities Model Program for Seniors (CHAMPS) physical activity questionnaire. METs were assigned specifically for older adults.^24^ CHAMPS activity has been validated against lower body strength,^24^ walk tests,^24^ and energy expenditure,^25^ although CHAMPS has also been uncorrelated with accelerometer measurement in special population such as fibromyalgia.^26^ Self-reported physical activities were coded according to five categories (Jurca et al.; Table 1).^15^ The categories were operationalized in this study as shown in Table 2.

**Table 2.** Operationalization of Physical Activity Categories

Physical activity category	Number of days of activity type (CHAMPS)	Number of hours of activity (CHAMPS)	Sample activities
1	Less than 5 anaerobic and 0 aerobic	—	Housework, light gardening, golf (cart), stretching, tai chi, yoga
2	5 or more anaerobic and 0 aerobic	—	
3	4 or fewer aerobic	At least 3 hours in one aerobic activity	Dance, tennis, running, brisk walking, bicycling, swimming
4	More than 4 aerobic	At least 3 hours in one aerobic activity	
5	Any number of multiple aerobic	At least 3 hours each in more than one aerobic activity	

#### Diary physical activity

On each diary day, women listed physical activities on that day that increased their heart rate or made them sweat. MET values from the 2,000 Compendium of Physical Activities^27^ were assigned to activities by three coders (Cronbach's α = 0.92; intraclass correlation = 0.79), and the average across raters was used to define activity type. Four women did not complete the physical activity section of the diary. For anaerobic activity (categories 1 and 2; METs between 3 and 4), the largest percent of women reported such an activity on 1 day (36%). For aerobic activity (categories 3–5, METs above 4), the largest percent of women again reported 1 day of such an activity (51%). Because all women reported at least 1 day of aerobic activity, the definitions in Table 2 were modified slightly to allow 1 day of aerobic activity in categories 1 and 2, and categories 1 and 2 were combined to stabilize the reference category. Diary and retrospective activity reports were essentially unrelated as categories (Spearman ρ = −0.01).

### Data analysis

eCRF was calculated using the weights provided by Jurca et al. and regressed on predicted CRF (pCRF) operationalized as predicted VO_2_max using measurements obtained from submaximal treadmill testing (pCRF).^15^ A second model regressed the model components individually on pCRF, thereby allowing the weights to be fit to the sample. These two models were each estimated twice, once with retrospective physical activity categories (CHAMPS) and once with diary physical activity categories.

## Results

The mean ± standard deviation (SD) of the predicted maximal HR achieved during the submaximal GXT was 86.2% ± 6.2% when a sex-specific predictive maximal HR equation was employed.^11^ Although every effort was made to encourage each participant to achieve a test termination HR as close to the upper limit of 150 bpm, as specified by the protocol used, low RHR and volitional fatigue prevented 22 (11.7%) of the participants from achieving 80% of their predicted maximal HR at test termination. The predicted maximal HR achieved for these 22 individuals ranged from 74.4% to 79.2% (mean = 77.2%, SD = 1.6%). A sensitivity analysis excluded participants with RHR >100 bpm (*n* = 3) or a submaximal termination HR of <115 bpm (*n* = 5). There was minimal change in the results; the full results of the sensitivity analysis can be found in the Supplementary Table S1.

**FIG. 1.**
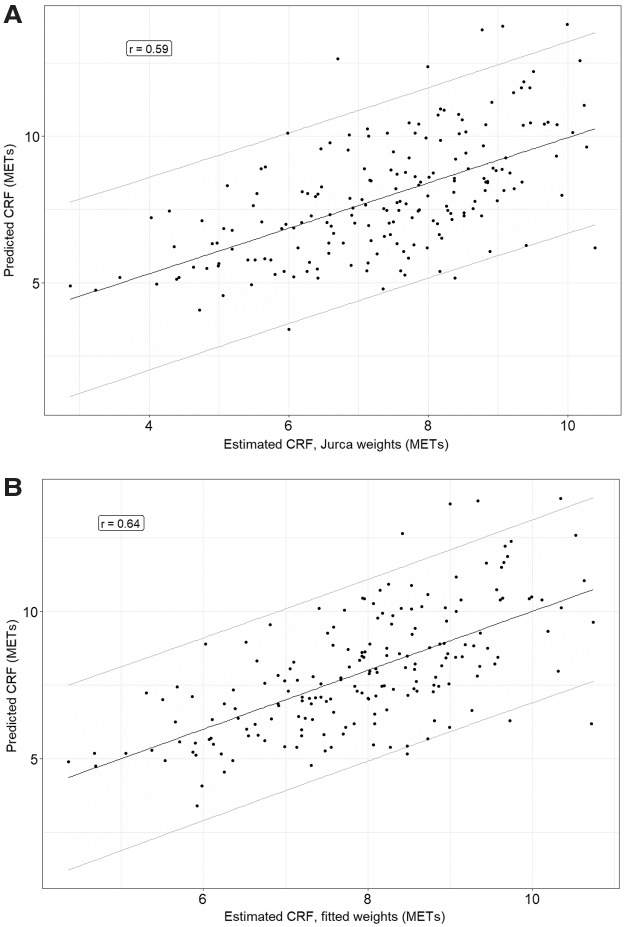
Plots of eCRF versus pCRF using assigned weights **(A)** or fitted weights for the sample **(B)**, with the regression line, 95% prediction interval, and correlation coefficient. eCRF, estimated cardiorespiratory fitness; pCRF, predicted cardiorespiratory fitness.

Table 3 shows the model results using the weights assigned by Jurca et al. and weights estimated on the sample.^15^ The beta weight for the regression of pCRF on eCRF was 0.77 (standard error [SE] = 0.08, *p* < 0.0001). The model using assigned weights accounted for 35% of the variance in pCRF; the model using sample-specific weights (Table 3) accounted for 42%. Assigned weights and estimated weights were similar for age, BMI, and RHR. However, the estimates for physical activity categories were different; there was little differentiation between categories 1 and 2, and higher categories contributed less to estimation of CRF as in the samples on which the assigned weights were developed. Figure 1 shows the scatter plots of pCRF against eCRF using assigned weights and fitted weights. Residuals from the model with fitted weights ranged from −4.6 to 4.5 (SD = 1.6).

**Table 3.** Model Weights When Assigned, Fitted to the Sample Using the CHAMPS to Assign Physical Activity Level, and Fitted to the Sample Using the Diaries to Assign Physical Activity Level

Variable	Weight from Jurca et al.	Weight estimate (SE) fitted to sample (CHAMPS)	Weight estimate (SE) fitted to sample (diary)
Intercept	18.07	21.2	22.7
Age	−0.10	−0.10^a^ (0.02)	−0.10^a^ (0.02)
BMI	−0.17	−0.21^a^ (0.02)	−0.22^a^ (0.02)
RHR	−0.03	−0.03^b^ (0.01)	−0.04^b^ (0.01)
Physical activity category			
1	0.00	0.00	0.00
2	0.32	0.03 (1.23)	0.00
3	1.06	0.67 (1.16)	0.34 (0.25)
4	1.76	0.76 (1.16)	−0.42 (0.53)
5	3.03	1.17 (1.13)	0.81 (0.82)
Model R^2^	0.35^a^/0.32^a,^^*^	0.42	0.40

Weights are unstandardized beta weights.

^a^ *p* < 0.0001, ^b^*p* = 0.002, ^*^CHAMPS/diary.

SE, standard error.

When diary physical activity categories were substituted for CHAMPs categories, there was little improvement in model fit either when assigned weights were used (*b* = 0.91, SE = 0.10, *p* < 0.0001, *R*^2^ = 0.32) or when fitted weights were used (Table 3; *R*^2^ = 0.40).

## Discussion

We have demonstrated the utility of routinely collected information for estimating CRF in midlife and older women. In this study, predicted VO_2_max was calculated from VO_2_ and heart rate measures during submaximal treadmill testing. Estimated VO_2_max was calculated using the previously validated weights reported by Jurca et al., which were derived from 2% submaximal, 94% symptom-limited maximal, and 4% maximal treadmill testing.^15^ Fitted VO_2_max was calculated from sample-specific weights. Both the estimated and fitted models performed well, accounting for 36% and 42% of the variance, respectively. The Jurca et al. eCRF equation performed well, but not as well as in more diverse samples, in which it predicted 56%–58% of the variance in CRF.^15^ Restriction of range in gender (women only), age (50–75 years), a relatively healthy cohort in this sample, and the use of a different GXT protocol may have contributed to the lower amount of variance accounted for. However, the influences of age, BMI, and resting HR were similar in this sample compared with the larger and more diverse samples used by Jurca et al. Examination of residuals suggested that both the estimated and fitted equations may have been more accurate at lower levels of fitness.

Objectively measured and eCRF are strong predictors of mortality in asymptomatic individuals as well as those with known metabolic and cardiovascular disease.^1,4–6,11,12^ Previous studies have estimated that for women, objectively measured METs of 7–8 were associated with *a* ≥ 50% reduction in mortality risk.^6,11,21,28,29^ Our sample had an average predicted MET of 7.9 with a range of 3.4–13.8 based on submaximal VO_2_ measurements, confirming a wide range of fitness in this cohort of women.

In our study, age, BMI, and resting HR were significant variables in the model, but the two different self-reports of physical activity did not contribute. This contrasts with several published reports in which both subjective and objectively measured physical activity were significantly positively associated with CRF in a variety of adult populations.^30–35^ The high frequency of the most active category using the CHAMPS suggested a higher level of fitness, but the least active categories had the highest frequency in the daily diaries. These measures were essentially unrelated as categories and are likely to capture different information. The CHAMPS is a directed, standardized instrument with physical activity assignments based on MET values that are both well established and modified to more accurately assess the physical activities of older and/or more sedentary adults. However, the CHAMPS relies on recall, which can be biased, whereas the daily diary was an open-ended, unstandardized instrument, but was collected closer in time to the activity itself.

The association between amount and intensity of total physical activity and CRF is relatively weak in general populations, accounting for only part of the variance in VO_2_max.^1,36^ However, another potentially important limitation of the study is the lack of objectively measured physical activity such as actigraphy. Objective and subjective measures of physical activity give qualitatively similar results for gender and age patterns of activity in children; however, accelerometer-measured activity was substantially lower than self-reported physical activity.^37^ The discrepancies between subjective and objectively obtained physical activity measures are not limited to child cohorts. Despite a significant relationship between subjective and objective physical activity measures, physical activity diaries reported significantly greater physical activity compared to objective physical activity monitors in a small cohort of older (>50 years of age) Filipino American women.^38^ In a cohort of older adults, despite a significant correlation among physical activity measures, the subjective physical activity questionnaire underestimated sitting and overestimated time spent in nearly all physical activity intensities.^39^ In our model, although the level of “actual” physical activity would expectedly be different from self-report, the relative contribution to the model would likely be similar because objective and subjective physical activity correlate.^40^ Which of the self-report measures (structured, retrospective or unstructured, contemporaneous) would best correlate with objective activity is a direction for future research.

Other limitations of the study include the cross-sectional design, which limits our ability to determine the effects of CRF on subsequent clinical outcomes. In addition, the sample is relatively small and homogenous, limiting generalizability to more diverse samples. Nevertheless, even within a cohort of women with restricted range in age (midlife and older), ethnicity (white, non-Hispanic), and health (stringent inclusion criteria), a regression-based estimate of CRF could explain sufficient variance to suggest that this method of estimating CRF is robust. Finally, we did not measure maximal or peak VO_2_, but calculated the VO_2_max using a submaximal graded exercise protocol.

Although the estimated models were related to the predicted VO_2_max, there was substantial variance in pCRF not accounted for in this sample. As a consequence, the 95% prediction intervals (the range in which a future prediction would be expected to fall; see Fig. 1) were wide. The 95% prediction interval was approximately ±3 METs. However, if one were able to tolerate a 75% prediction interval, that range drops to approximately ±1.8 METs, and for 50%, approximately ±1 METs. Future research should identify additional determinants of CRF in older women to improve prediction precision.

## Conclusions

The results of this study suggest that there is a relationship between variables that are easily obtained from clinical data (age, BMI, resting HR, and physical activity) that can potentially estimate CRF. Even in small, homogeneous samples, the regression method of estimating CRF as proposed by Jurca et al. appears to be valid, and women with a low eCRF may benefit from more intensive counseling on CRF, enhancing physical activity.^14,41^

## Supplementary Material

Supplemental data

## Abbreviations Used

ACLS: Aerobics Center Longitudinal Study
ADNFS: Allied Dunbar National Fitness Survey
BMI: body mass index
CRF: cardiorespiratory fitness
DAHLiA: Daily Activity and Health in the Lives of Adult Women
ECG: electrocardiogram
eCRF: estimated cardiorespiratory fitness
GXT: graded exercise test
HR: heart rate
MET: metabolic equivalent
NASA: National Aeronautics and Space Administration
pCRF: predicted cardiorespiratory fitness
RHR: resting heart rate
SD: standard deviation
SE: standard error
