# Supplementary material for: Estimation of Cardiorespiratory Fitness Without Exercise Testing: Cross-Validation in Midlife and Older Women
Source: Womens Health Rep (New Rochelle). 2020 Dec 28;1(1):584–91. doi: 10.1089/whr.2020.0045 (PMC9902048; doi:10.1089/whr.2020.0045)
Supplement: Supplemental data [file Supp_TableS1.docx]

| **Sensitivity analysis.** Model weights when assigned, fitted to the sample using the CHAMPS to assign physical activity level, and fitted to the sample using the diaries to assign physical activity level. *Eight observations excluded for RHR > 100 or termination HR < 115.* | | | |
| --- | --- | --- | --- |
| **Variable** | **Weight**  **from Jurca et al. (2005)** | **Weight estimate (SE) fitted to sample (CHAMPS)** | **Weight estimate (SE) fitted to sample (diary)** |
| Intercept | 18.07 | 21.2 | 22.9 |
| Age | -0.10 | -0.09 (0.02) | -0.10 (0.02) |
| BMI | -0.17 | -0.21 (0.03) | -0.22 (0.03) |
| RHR | -0.03 | -0.04 (0.01) | -0.04 (0.01) |
| Physical activity category |  |  |  |
| 1 | 0.00 | 0.00 | 0.00 |
| 2 | 0.32 | 0.06 (1.23) | 0.00 |
| 3 | 1.06 | 0.66 (1.17) | 0.28 (0.25) |
| 4 | 1.76 | 0.75 (1.17) | -0.47 (0.54) |
| 5 | 3.03 | 1.12 (1.14) | 0.77 (0.83) |
| Model R^2^ | 0.35/0.30* | 0.41 | 0.40 |
